# Improvement and Evaluation of a Device That Determines the Interfacial Shear Strength of Carbon Fiber/Polyphenylene Sulfide Composites

**DOI:** 10.3390/polym15183749

**Published:** 2023-09-13

**Authors:** Yuan Dong, Jia-Cao Yang, Xiao-Jun Wang, Gang Zhang, Mei-Lin Zhang, Zhi-Mei Wei, Sheng-Ru Long, Jie Yang

**Affiliations:** 1 College of Polymer Science and Engineering, Sichuan University, Chengdu 610065, China; doyuan95@163.com; 2 Analytical and Testing Center, Sichuan University, Chengdu 610064, China; 3 State Key Laboratory of Organic–Inorganic Composites, Beijing 100029, China; 4 State Key Laboratory of Polymer Materials Engineering, Sichuan University, Chengdu 610065, China

**Keywords:** improved apparatus, microbond test, interfacial shear strength, carbon fiber, polyphenylene sulfide

## Abstract

This study improved homemade apparatus for characterizing the interfacial shear strength (IFSS) of carbon-fiber-reinforced polyphenylene sulfide (PPS/CF) composites. The upgraded generation II experimental device includes a newly developed experimental clamp for samples, as well as testing systems. Compared with the initial generation I apparatus and the commercial Toei instrument, the generation II device is easier and more efficient to operate. The average interfacial adhesion values obtained using these devices were consistently approximately 40 MPa, with relatively low data scatter, showing excellent repeatability and applicability during microbond tests. Notably, the generation II experimental device was equipped with an additional high-frequency data-capturing tool to identify the debonding peak force more precisely, which demonstrated a higher interfacial shear strength of 42.81 MPa during testing. Therefore, the new instrument was able to reflect the change in the interfacial stress state during the interface debonding process more accurately and reliably.

## 1. Introduction

As a result of their good thermal stability, as well as their high strength and modulus, carbon-fiber-reinforced polymer composites have been widely applied in many fields, including aircraft construction, automotive industries and transportation [1,2,3,4,5]. It is widely acknowledged that composites’ mechanical properties depend not only on the resins’ intrinsic material properties, but are also significantly influenced by the interfacial region, which is responsible for transferring external stress from the matrix to the reinforced fiber [6,7]. Thus, it is of great importance to characterize the interfacial status of composites using quantitative evaluation parameters [8]. Researchers have proposed many micromechanical test methods to evaluate interfacial properties, including fragmentation [9,10], push-out [11,12], pull-out [13] and microbond tests [14,15,16]. Among these experimental approaches, the microbond test has recently attracted significant attention from researchers, due to its simple procedure and versatile applicability.

In the microbond test, a resin matrix microdroplet is pulled out of a single reinforcing fiber using two parallel blades. During the pull-out process, the tensile force increases continuously until interfacial debonding between the fiber and the resin occurs, reaching the maximum pull-out force. Various parameters, encompassing the fiber diameter, the embedded length and the debonding peak force, can be quantified to evaluate the interfacial status. A critical aspect of this test is that the embedded length of the matrix in the fiber should be controlled within the critical length. This requirement ensures that the sample’s maximum tensile load does not exceed the monofilament carbon fiber’s critical tensile load, to prevent fiber breakage before debonding. Consequently, microdroplets’ sizes need to be well controlled. Furthermore, Sockalingam et al.’s [17] comprehensive review summarized other factors that impact measurements. Even a slight alteration in the separation distance between the fiber and the blades’ edges could result in significant variations in the magnitude and location of peak shear stress along the interfacial direction [18]. The results of both microbond tests and finite element analyses demonstrated that the distance between the knives and the fiber was positively correlated with interfacial debonding when the interfacial adhesion was within a certain range [19]. Warrior et al. [20] also considered the influence of the droplet’s morphology and the fiber’s diameter on experiments. Force–displacement curves and IFSS results from a finite element simulation model were verified using experimental data. Next, calculation results from the finite element model indicated that the meniscus of droplets had a negligible impact on the average value. However, an elongated meniscus could cause variation in stress distribution at the contact location and its surrounding area, potentially affecting the failure mode of the microdroplet model and compromising the reliability of the final test results. Additionally, minor discrepancies in the diameters of individual fibers could significantly influence the IFSS determined using microbond tests, particularly for composites with poor interfacial bonding properties. Consequently, to achieve consistent and reliable experimental results, it is crucial to solve these issues and maintain accurate and constant geometric parameters during comparison experiments. Moreover, the experimental apparatus’ limitations also need to be considered; testing demands precise micromechanical equipment. Although the microbond test is commonly employed, there has been little standardization of preparation methods for samples and tensile instruments [14]. Thus, most individual laboratories tend to design their own equipment by applying the same theory; however, the comparison of experimental results between different instruments is rarely mentioned. Craven et al. [21] reported that their test was carried out using a universal Instron tensile tester (Model 1026) configured with a 0.5 N cantilever load cell to identify the adhesion of silk/epoxy composites. Two general parallel stainless steel blades were installed on the instrument to pull out microdroplets. Concurrently, an image of each sample was individually captured using a video camera connected to the computer’s software. Similarly, a commercial Instron 5567 testing system was also employed by Zhi [22] for microbond testing, and a pair of tailor-made adjustable blades was improved and mounted on a miniature camera to measure more flexibly. To enhance the test’s efficiency, Wang [23] designed a special fiber-gripping system for a micro-tensile device to measure CF/epoxy composites based on an Instron 5848 mechanical tester. The fiber clamps with slots could more effectively grip microdroplets’ short fibers to pull out samples stably and reduce the breakage caused by fiber defects during testing. Additionally, the instrument’s imaging system was combined with the test system to realize observation of the full testing process. Recently, Laurikainen et al. [24] designed high-precision blades and developed a computer vision algorithm to automatically identify sample dimensions, and thus realize the equipment’s high-throughput capabilities. Although the micro-tensile instruments developed by researchers over the years have mainly been applied to thermosetting composites, they may not be suitable for experiments on thermoplastic composites [21,25,26], which require different sample preparation procedures and may have different fiber lengths or interfacial bonding statuses [27,28].

Several laboratories have attempted to develop devices for thermoplastic composites [29,30,31]. In previous work, our team proposed a corresponding homemade device [32], including knife blades and CCD cameras, as well as a clamping system, displacement gauge, actuator, load cell, heating chamber, etc. However, the equipment still requires improvement. For instance, samples could not be tightly clamped using the flat clip and could not be clearly observed during the debonding process. Additionally, the blades’ movement process was unstable, and it was difficult to accurately control samples’ temperature during testing. Moreover, the influence of factors related to different instruments and test parameters on measurements of interfacial shear strength have not been fully discussed.

In this study, we aimed to improve our original device based on previous studies in order to perform microbond tests more simply and accurately. The apparent interfacial shear strength ($\tau_{app}$) of carbon-fiber-reinforced polyphenylene sulfide composites obtained using the generation II instrument was compared with test results determined using the generation I instrument and the commercial device. PPS/CF composites have high mechanical properties and stable chemical properties, which make them suitable for investigating the repeatability and stability of the microbond test method [33,34,35,36].

## 2. Design and Manufacture of the Generation II Experimental Apparatus

**Figure 1:**
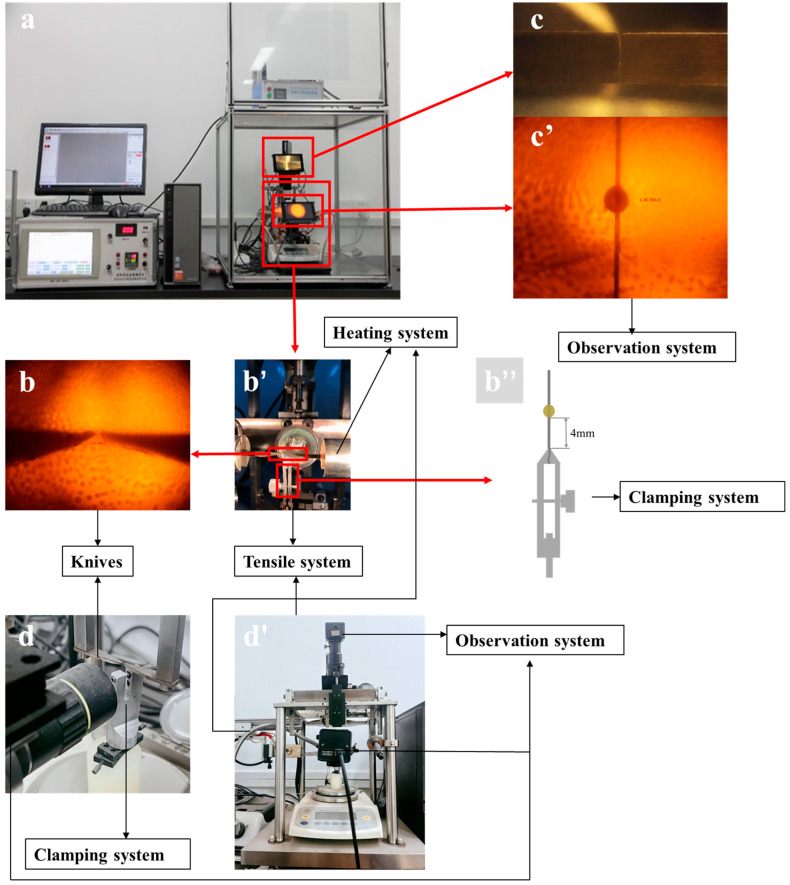
(**a**) An overview of the generation II experiment apparatus; (**b**) knives of the tensile system, (**b′**) the tensile system and (**b″**) schematic diagram of the clamping system; (**c**,**c′**) vertical and horizontal observation systems, respectively; (**d**) partial view and (**d′**) overview of the generation I experimental apparatus.

As illustrated in Table 1, the generation II apparatus’ improvements upon the generation I device [32] include updated clamping, observation, tension and heating systems. These improved systems increased the efficiency and accuracy of the operation process; overviews of both devices are illustrated in Figure 1. The curved clip, presented in Figure 1b″, was specifically manufactured to firmly clamp the bottom of the fiber during the measurement process, and was more reliable than the generation I device’s flat clip, shown in Figure 1d. To more precisely control the stretching process, a pair of individually movable stainless steel blades (Figure 1b,b′) was meticulously designed to pull out the droplet. These blades could be individually moved with an accurate resolution of 0.05 μm through the integration of optics devices (Figure 1c,c′) to decrease the blades’ gap. Compared to the generation I device’s blades, which could only be moved on the horizontal x-axis, the improved generation II blades could be adjusted three-dimensionally. The blades’ movement was actuated by an SC0 servo motor (Times Brilliant Co., Ltd., Beijing, China) using a DM-055B controller (instead of the stepper motor actuator used in the generation I apparatus) to enhance the stretching process’ stability and the stretching rate’s adjustability. A high-sensitivity electronic balance (0.001 g resolution, Sartorius BSA423S, Goettingen, Germany) was modified and secured in the tensile system to record external forces during testing and transfer them to the testing software. Data acquisition was set to record signals with a 10 Hz frequency. In contrast to the generation I instrument’s clamping system, which was in contact with the balance’s plane (Figure 1d′), the generation II instrument’s sample clamping device was tightly mechanically riveted to the balance’s internal structure. The test fixture used to hold the sample was securely screwed by the clamping system, ensuring a highly sensitive transmission of tensile forces. The force’s accuracy was closely related to the balance sensor’s precision, and the force was usually calibrated using the balance. Moreover, the parameter of microdroplets and the knife tools’ distance could be captured accurately using zoomable microscopes (XZ-2B, Ningbo Yongxin Optics Co., Ltd., Ningbo, China) (Figure 1c,c′), which were connected to a computer running customized imaging software (Andonstar Measure software 3.2.0.0, Shenzhen, China). The sample testing process could also be recorded online using the observation system to display crack initiation and propagation in a fiber–matrix system, whereas the observation of a sample’s dimensions and the measurement process were separated using the generation I equipment. In addition, a heating and cooling system with 0.1 °C temperature control precision was installed on the knives, which allowed the temperature around the specimen to be regulated more accurately than it was using the generation I device’s heating system. These improvements will be useful for future experiments related to composites’ temperatures.

## 3. Experimental Methods

### 3.1. Materials

Custom-built carbon fibers with a ~7 μm diameter (without sizing treatment) were supplied by Xingke Carbon Fiber Co., Ltd. (Dalian, China); their average tensile strength and elastic modulus were 3.5 GPa and 230 GPa, respectively. Polyphenylene sulfide resin (PPS), provided by Japan Polyplastics Co., Ltd. (Tokyo, Japan), was the thermoplastic resin matrix used in this study.

### 3.2. Sample Preparation

**Figure 2:**
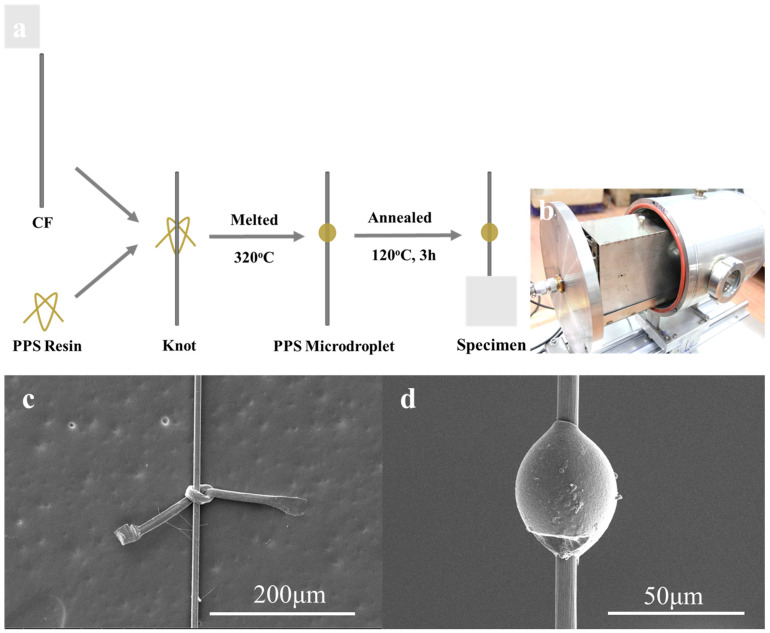
(**a**) Procedure to prepare PPS microdroplet on the CF fiber glued onto a small card and (**b**) the vacuum melting device. SEM images of samples: (**c**) the microdroplet before melting and (**d**) the fiber surface after debonding.

This study’s preparation method was based on work proposed by our group as suitable for thermoplastic resin [32]. As demonstrated in Figure 2a, the PPS resin was first melted at 320 °C (above its approximate 280 °C melting point) and quickly pulled into the PPS resin fiber. The resin fiber was knotted around a single carbon fiber and slightly tightened; then, the extra resin fiber on the CF was cut off. For ease of testing, the carbon fiber was previously glued onto a small card and the free fiber length was maintained at approximately 10 mm. The diameter of the resin fiber selected was close to the diameter of the carbon fiber monofilament to control the microdroplet’s embedded length. Next, within a few minutes, the specimen was completely melted at 320 °C in a melting device to form a microdroplet, and then, quickly quenched in air.

Notably, resins’ chemical and physical properties may change during the melting process. In particular, resins with low melting points are susceptible to oxidation and degradation. In Thomason et al.’s [37] research, thermal degradation of the matrix during sample fabrication resulted in variations mechanical properties, reducing composites’ interface adhesion. To prepare samples with a stable interfacial state, a customized vacuum melting device (Figure 2b) was designed for the generation II instrument. Samples were secured to a rack, which was then placed in a heating tank. Four infrared heating rods were vertically installed on both sides of the heating tank. The infrared heating rods quickly heated up; they reached the resin’s melting temperature in a short time. Adding a metal cover around the heating rod concentrated the heat. The melting unit’s outer section was the vacuum box, which was mechanically fixed with screws at the joints. During the melting process, the rack containing the samples was first placed into a heating bath, and then, enclosed with a metal cover. Subsequently, the vacuum chamber was tightly sealed, and the air within the device was evacuated using a vacuum pump. The temperature was rapidly increased from room temperature to the 320 °C melting temperature to melt the microdroplets. The sample was removed immediately after melting to avoid prolonged exposure to high temperatures. Images of the samples before after and debonding were examined using a scanning electron microscope (Quanta 250, FEI, Hillsboro, OR, USA) and are displayed in Figure 2c,d, respectively. It was observed that the sample knotted with the PPS resin fiber around a carbon monofilament formed a symmetrical shuttle microdroplet after the melting treatment.

Finally, to remove the thermal history, each microdroplet was annealed at 120 °C for 3 h and slowly cooled to room temperature. Only well-shaped, axisymmetric microdroplets were chosen for testing under the microscope. This specimen-forming procedure was used for generation I and generation II microbond device testing.

**Figure 3:**
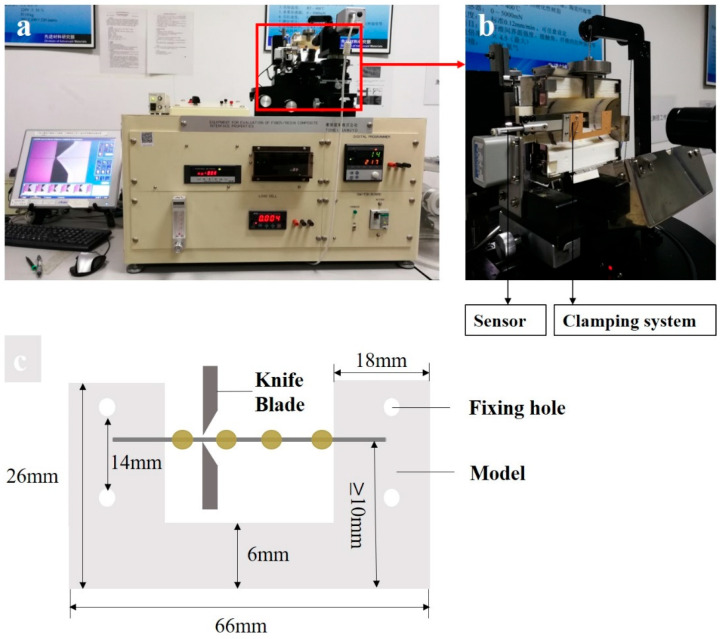
(**a**) An overview of the Toei device; (**b**) the partial testing system; and (**c**) a schematic diagram of the template for the microbond test using the Toei tester.

The comparison experiment of interfacial properties used commercial interfacial evaluation equipment [34], which was supplied by the Suzhou Institute of Nano-Tech and Nano-Bionics (SINANO), Chinese Academy of Sciences. As revealed in Figure 3a, the commercial micro-tensile experiment instrument was manufactured using Equipment for Evaluation of Fiber/Resin Composite Interface Properties (model HM410, Toei Sangyo Co., Ltd., Tokyo, Japan). The preparation procedure for PPS/CF composite samples was similar to the method described above, except that the CF length required adjustment for the specific test template. The sample was glued in a standard template, and then, clamped to the stretching device shown in Figure 3b. The stress resulting from the vertical knives stretching the PPS resin microdroplet was measured using the sensor and transmitted to the software. The standard model parameters and the sample preparation standard are shown in Figure 3c. Notably, to ensure consistency of the samples’ interfacial states, the microdroplets’ preparation method should remain consistent with the previous work. Typically, more resin microdroplets should be prepared on the single carbon fiber of the test template to improve experimental efficiency.

### 3.3. Experimental Procedure

**Figure 4:**
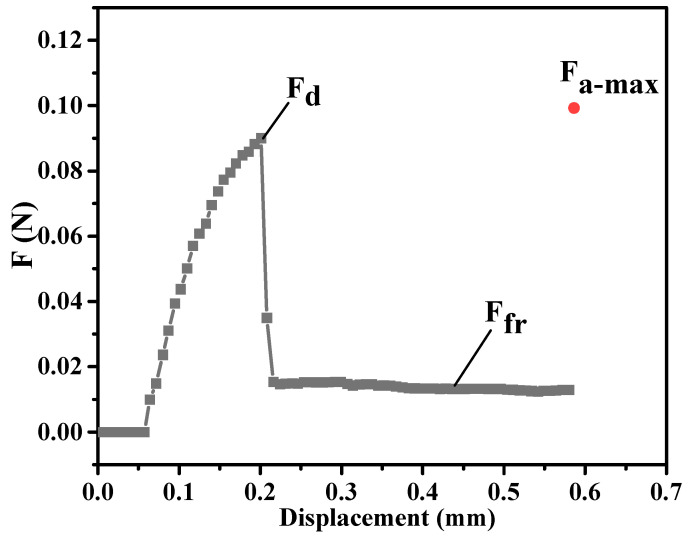
The load–displacement curve of PPS/CF composites obtained from the microbond test using the generation II device. (There was another absolute maximum value ($F_{a-max}$) besides the maximum force ($F_{d}$), due to the additional data-capturing tool.)

One end of the sample, as indicated in Figure 1, was gripped tightly in the axis direction using a special clamp and was attached via a steel pillar on the load cell. With the help of the horizontal microscope, a clear image of the sample could be observed, as shown in Figure 1c′. The embedded length of the microdroplet and the diameter of the fiber were both clearly determined using the observation system before the pulling test was carried out. Subsequently, the two parallel blades on either side of the sample were adjusted to position the microdroplet above the knives, which were gradually narrowed until the slit width reached approximately 10~20 μm. The tensile system moved upward, with a constant movement rate of 0.02 mm/min, to contact the microdroplet, and the load was measured using a chart recorder. When external force was applied to the resin, the load began to gradually increase until the shearing force exceeded the interfacial shear strength of the composites; then, the debonding process occurred, resulting in a sharp decrease in the load from the peak force. The load decreased to a constant sliding friction force ($F_{fr}$) with a continuous slight fluctuation, and the microdroplet continued to slide along the free length of the fiber. The normal maximum pull-out force ($F_{max}$) could be obtained through the sharp peak force of the force–displacement curve displayed in Figure 4. Moreover, a maximum load catcher with a high recording frequency was added to the generation II instrument, and there was another absolute maximum value ($F_{a-max}$) besides the maximum force ($F_{d}$) displayed in the curve. Combined with the embedded length of the microdroplet and the fiber diameter, the apparent interfacial shear strength (IFSS, $\tau_{app}$) can also be calculated according to Equation (1) [32]; the average IFSS was obtained from test data of approximately 40 specimens.

(1) $$\tau_{app}=\frac{F_{max}}{\pi d_{f}l_{e}}$$

Here, $F_{max}$ is the maximum pull-out force and involves $F_{a-max}$ and $F_{d}$; $d_{f}$ is the fiber diameter; and $l_{e}$ is the embedded length of the microdroplet. The experimental procedures employed using the generation I and Toei testers were carried out using the research methods of Liu [32] and Thomoson [30], respectively.

### 3.4. Comparison of Instruments

The comparison results for generation I and generation II devices and the Toei tester are shown in Table 2, and reveal that the generation II device’s observation and test systems were better than those of its counterparts. The highest-resolution image was obtained from the generation II tester; its apparatus was more efficient than the Toei tester’s. The generation II tester’s plane blades are suitable for all resins, whereas elastic resins might be squeezed out by blades with an obtuse angle surface. The commercial instrument’s sensor, shown in Figure 3b, needs to be stuck vertically into a fixed slot, which makes it easy to damage in practice. In addition, the three instruments’ recording frequencies were close, indicating the same accuracies during testing.

## 4. Results and Discussion

### 4.1. Samples’ Morphologies 

**Figure 5:**
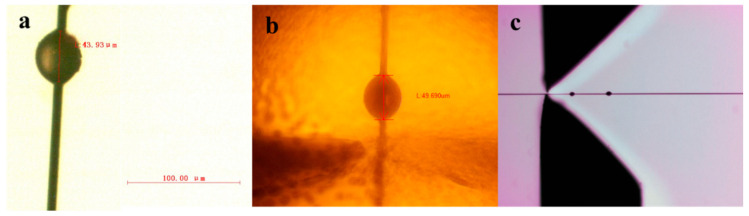
Real images of microbond samples captured using (**a**) the generation I device, (**b**) the generation II device and (**c**) the Toei tester.

The images of microbond specimens of similar size (Figure 5) indicate that the knotting method worked well in controlling the embedded length of PPS resin on the carbon fiber to form a symmetrical microdroplet. At the same time, pictures taken using the instruments’ microscopes displayed the samples’ actual status during the measurement process. The resolution and magnification of images obtained using the generation I and generation II devices (Figure 5a,b) were higher, so the samples’ embedded lengths could be more accurately determined. However, the image in Figure 5c demonstrates that the samples’ embedded lengths could not be accurately measured using the Toei tester; when the magnification increased, the image appeared so blurred that it was difficult to measure.

### 4.2. IFSS of PPS/CF Composites

**Figure 6:**
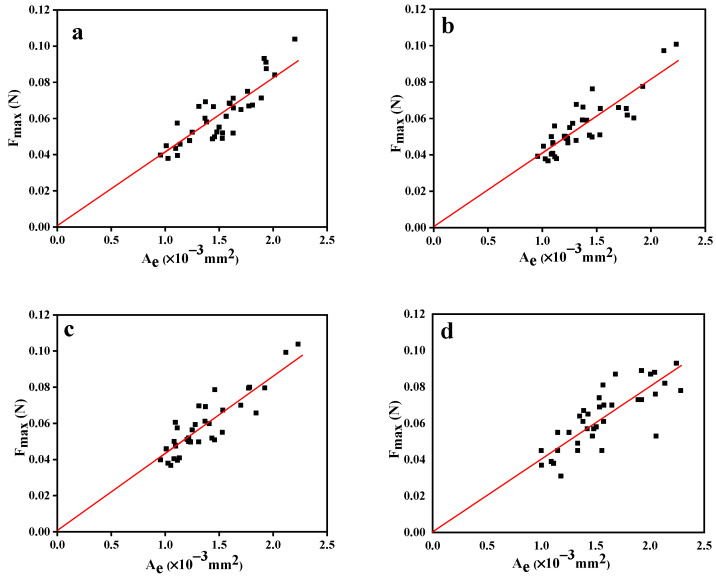
Plots of the maximum pull-out force as a function of embedded area: (**a**) the generation I device; (**b**) the generation II device (no catcher); (**c**) the generation II device (involved catcher); and (**d**) the Toei device.

Plots of the maximum force vs. embedded area for samples evaluated using different devices are displayed in Figure 6. As a result of the annealing procedure to eliminate the influence of residual thermal stress, the data’s fitted straight lines pass the origin points, and slopes obtained from the regression lines reflect the apparent interfacial shear strength of the PPS/CF composites [36]. The scatter plots also show that the embedded fiber length of microdroplets prepared using the knot-melt method were controlled within a certain range, and each sample’s data are evenly distributed on both sides of the regression line, showing relatively stable test results.

**Figure 7:**
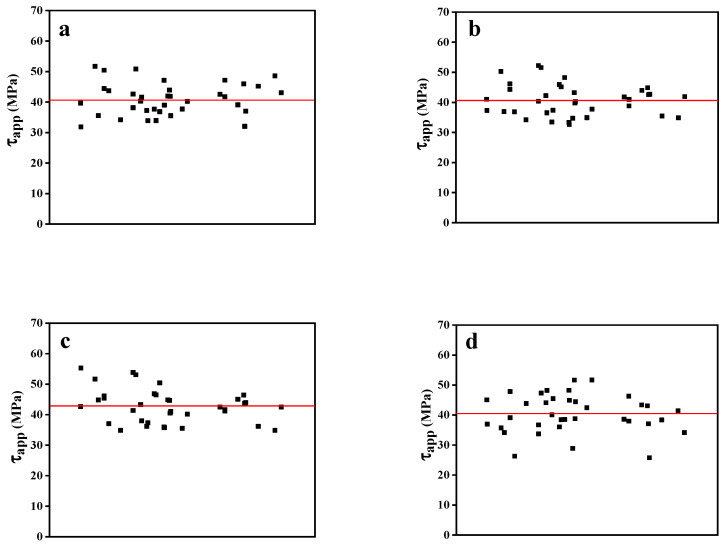
The average values of interfacial shear strength: (**a**) the generation I device; (**b**) the generation II device (no catcher); (**c**) the generation II device (involved catcher); and (**d**) the Toei device.

Meanwhile, according to Equation (1), the average value of $\tau_{app}$ can be calculated using the individual IFSS of each sample based on the maximum force as a function of the embedded area, whose mean values and IFSSs obtained using the slope of the fitting line are both displayed in Figure 7 and Table 3, reflecting close results. These results demonstrate that the IFSSs obtained using different devices were all approximately 40 MPa, and the degrees of data dispersion were relatively similar. This implies that these sets of instruments have great data reliability and comparability. Moreover, due to the generation II instrument’s maximum-value capture device, its load sensor was more sensitive to changes in force, resulting in a larger recorded absolute maximum force. Consequently, the final interfacial shear strength of the generation II device was 42.81 MPa, surpassing the values obtained using other devices. This study’s results suggest that obtaining the maximum value can make the test results better reflect the real-world situation. In addition, the microdroplet’s size was difficult to determine using the Toei tester, which affected the test results’ accuracy and caused a significant degree of dispersion of the overall test results. Therefore, our results illustrate the successful improvement of the instrument and prove the success and universality of the microbond test.

## 5. Conclusions

In this study, we introduced an improved generation II experimental device based on the generation I apparatus, designed specifically for microbond testing to evaluate the interfacial shear strength of carbon-fiber-reinforced PPS matrix composites more accurately. Instead of using the universal tensile tester, the instrument was custom-built; the designed system combined samples’ measurement and tensile processing online together, which was convenient to adjust, efficient to test and suitable for almost all polymer composites.

Moreover, comparative tests were conducted using three different instruments to investigate the repeatability and stability of the microbond test method. The results for the measured PPS/CF samples’ average interfacial shear strength, calculated using the tensile curves of different devices, were approximately 40 MPa, indicating the excellent repeatability of microbond tests and the stability of the experimental values. However, the generation II device’s added maximum load catcher accurately recorded the load with a high frequency; compared with the generation I device and the Toei tester, its interfacial shear strength measurement improved to 42.81 MPa, which was closer to the material interface strength’s true value. This illustrates the successful improvement of the instrument and that it can accurately reflect composites’ stress states; it also proves the success and universality of the microbond test.

Notably, there is still no unified sample preparation standard or corresponding measurement equipment for microbond testing. The improved equipment presented in this study has been successfully applied in thermoplastics tests [38,39]. The improved microbond test apparatus is also currently used in many research institutions. Therefore, this study is significant in promoting microbond method standardization for all polymer materials.

## Figures and Tables

**Table 1:** Parameters of generation I and generation II microbond devices.

Device		Generation I Device	Generation II Device
Clamping system		Flat clip	Curved clip
Observation system	Microscope	Free-zoom horizontal	Zoomable horizontal microscope
	Measurement	Separated from test	Combined with test online
Tensile system	Motor	Stepper motor	Servo motor
	Sensor	Sartorius BSA323S	Sartorius BSA423S
Heating system	Temperature	RT-160 °C	−20–300 °C

**Table 2:** Parameters of different devices.

Device		Generation I Device	Generation II Device	Toei Device
The magnification of microscopes (max)		160X	300X	4.5X
Blades		Plane	Plane	Obtuse angle surface
The test process	Direction	Vertical	Vertical	Horizontal
	The range of pulling rate	0.01–1 mm/min	0.001–2 mm/min	0.12 mm/min (default)
Collection of data	precision	0.001 g	0.001 g	0.01 mN
	Recorded frequency (s)	0.1	0.1	0.125

**Table 3:** Statistical results of IFSS for PPS/CF samples measured using different devices.

Device	$k_{e}$ (MPa)	$\bar{\tau_{app}}\;(\mathrm{MPa})$	STDEV
Generation I device	41.06	40.94	5.27
Generation II device (no catcher)	40.69	40.68	5.23
Generation II device (involved catcher)	43.01	42.81	5.54
Toei tester	40.18	40.41	6.36

## Data Availability

All the data are available in the paper.
